# The MacNew Questionnaire Is a Helpful Tool for Predicting Unplanned Hospital Readmissions After Coronary Revascularization

**DOI:** 10.14740/jocmr2447w

**Published:** 2016-01-26

**Authors:** Cesare Baldi, Renato De Vecchis, Carmelina Ariano

**Affiliations:** a Heart Department, Interventional Cardiology, A.O.U. “San Giovanni di Dio e Ruggi d’Aragona”, Salerno, Italy; b Cardiology Unit, Presidio Sanitario Intermedio “Elena d’Aosta”, ASL Napoli 1 Centro, Napoli, Italy; c Neurorehabilitation Unit, Clinica S. Maria del Pozzo, Somma Vesuviana (NA), Italy

**Keywords:** Coronary revascularization, Quality of life, Hospital readmission

## Abstract

**Background:**

The MacNew questionnaire is a neuro-behavioral tool which is easy and immediately usable. This self-reported questionnaire filled out by the patient allows the physician to achieve helpful information concerning the ways for optimizing the therapy and patient’s lifestyles. In this retrospective study, our aim was to assess whether relatively high scores found using the MacNew questionnaire in patients who had undergone percutaneous or surgical revascularization were associated with a decreased risk of unscheduled hospitalizations during the follow-up.

**Methods:**

A retrospective analysis concerning 210 patients was carried out. The clinical sheets of these patients were examined as regards the information provided in the specific questionnaires (MacNew Italian version) routinely administered during the hospitalization prescribed for recovering from recent interventions of coronary percutaneous or surgery revascularization. Every patient undergoing the psychological test with MacNew questionnaire was followed up for 3 years.

**Results:**

Using univariate analysis, a global score’s high value (i.e., above the median of the whole examined population) was shown to be associated with a significantly decreased risk of rehospitalization (HR (hazard ratio): 0.4312; 95% CI: 0.3463 - 0.5370; P < 0.0001). After adjustment for age, gender and myocardial infarction as initiating event, using a multivariate Cox proportional hazards regression model, the protection exerted by a high MacNew score against the risk of hospitalizations remained significant (HR: 0.0885; 95% CI: 0.0317 - 0.2472; P < 0.0001).

**Conclusions:**

A relatively elevated MacNew global score appears to be associated with a significantly decreased risk of unscheduled hospitalizations after coronary revascularization over a 3-year follow-up.

## Introduction

Examination of the quality of life through a self-reported questionnaire filled out by the patient is a way in which the psychological experience of the patient with heart problems can be explored and evaluated. In this regard, the MacNew questionnaire in its original version [1] is a diagnostic tool which consists of a number of questions answered on a seven-point scale. Among the multiple possible choices, each marked by a box, the examined patient will have to tick only one. In fact, more than one answer for each proposed question is not allowed, for the formulation of the final score. Global and subscale scores are computed by dividing the sum of the scores achieved for each individual item by the number of items listed in the questionnaire. The MacNew is a good example of successful interaction between psychologists and cardiologists in order to build a cognitive tool which is easy and immediately usable, able to effectively investigate in a quick manner the psychological problems of the patient with coronary heart disease (CAD) [2].

It has been successfully applied, especially in the field of cardiac rehabilitation to assess the psychological aspects underlying the psychophysical recovery phase subsequent to percutaneous or surgical revascularization in patients with CAD. Both for percutaneous revascularization and the surgical treatment (coronary artery bypass grafting (CABG)), there are a considerable proportion of patients (approximately 17% of patients according to optimistic estimates) [3] in whom the intervention does not achieve a satisfactory improvement in the quality of life. This is because it is followed by veiled or clinically obvious complications that will require the patient to undergo new hospitalization in the short or medium term.

The purpose of this study was to assess whether relatively high scores on the MacNew questionnaire are associated with a significant decrease in the risk of unplanned hospitalizations for cardiovascular causes during follow-up (3 years following the revascularization procedure).

## Methods

This study consisted of a retrospective analysis that involved 210 patients followed up for 3 years after a procedure of revascularization (coronary angioplasty with stent or CABG). According to customary practice conducted at the two institutes (Ed’A and SMdP) for cardiovascular rehabilitation involved in the study, all of the patients underwent an evaluation using the MacNew questionnaire, in the context of the practices of psychological and psychosomatic assessment that are routinely implemented in patients who are treated for recovering from recent interventions of coronary percutaneous or surgical revascularization. Additionally, every patient was requested to fill out a declaration of informed consent before the administration of the MacNew questionnaire.

The Italian MacNew explores 27 questions, including seven questions about symptoms, in three subscales that evaluate physical, emotional, and social function; a global health-related quality of life (HRQL) score can be computed from all scored items (Supplementary 1, www.jocmr.org). The retrospective evaluation was conducted through the analysis of the compiled questionnaires, stored as attachments within the medical records of the patients who had practiced clinical check-ups (Ed’A) or had undergone a planned period of cardiovascular rehabilitation (SMdP). Permission to retrieve and consult the patients’ records was obtained from the Hospital Directorates of the respective Institutes (Ed’A and SMdP), also considering the research purpose specified in the application for accessing the consultation of medical records. The anonymity of each patient was strictly maintained, in keeping with the current rules and regulations concerning privacy preservation. The time frame considered by the research involved the cardiovascular outpatient medical visits (Ed’A) as well as the planned admissions in day-hospital regimen for cardiovascular rehabilitation (SMdP), that had taken place during the years 2010 and 2011 after coronary revascularization procedures. Furthermore, the study was also extended to the subsequent clinical follow-up for an overall duration of 3 years. Patient demographic and clinical baseline characteristics were derived retrospectively from the outpatient clinical folders retrieved from the archives of the two centers actively engaged in the post-procedural diagnostic and therapeutic management of the patients. Follow-up data were also derived from hospital records of patients who were readmitted for any cardiac reason.

### Statistical analysis

All statistical tests were performed with a commercially available statistical analysis program (SPSS 15.0 for Windows, SPSS Inc., Chicago, IL, USA). Categorical variables were compared with Chi-square test, while the comparisons for continuous variables were made using the Student’s *t*-test.

To compare outcomes according to HRQL status, MacNew global scores above the median (50th percentile) were compared with those located below the median (lower quartiles). Moreover, separate logistic regression models were constructed for low/moderate (i.e., below the median) global HRQL score and high (i.e., above the median) global HRQL score as a single predictor variable. Statistical results were considered significant when P < 0.05 (two-sided). Also Kaplan-Meier curves were built by making a comparison between the global low-moderate and high HRQL scores, according to the respective definitions previously mentioned and by assuming the unscheduled re-hospitalization over a follow-up of 3 years as a relevant endpoint.

## Results

In the total cohort of 210 patients retrospectively enrolled, the mean MacNew HRQL scores were 4.38 ± 1.56 on the global scale, 4.46 ± 1.1 on the physical, 4.54 ± 1.1 on the social and 4.384 ± 1.5 on the emotional subscale. Median time delay between revascularization and administration of the questionnaire was 55 days (interquartile range 25 - 79 days). As previously stated, for analysis of rehospitalization, the MacNew global score was dichotomized into MacNew low score group (i.e., below the median, namely consisting of lower quartiles) and MacNew high score group (i.e., above the median, namely upper quartiles). The median of the MacNew global score was found to be 4.70. Additionally, among the various calculations that were performed, the following measurements were worthy of being reported: group with MacNew “low” score: median = 3.4 (min/max = 1.18/4.70); group with MacNew “high” score: median = 5.55 (min/max = 4.72/6.55).

### Relationship between HRQL scores and rehospitalization

**Figure 1:**
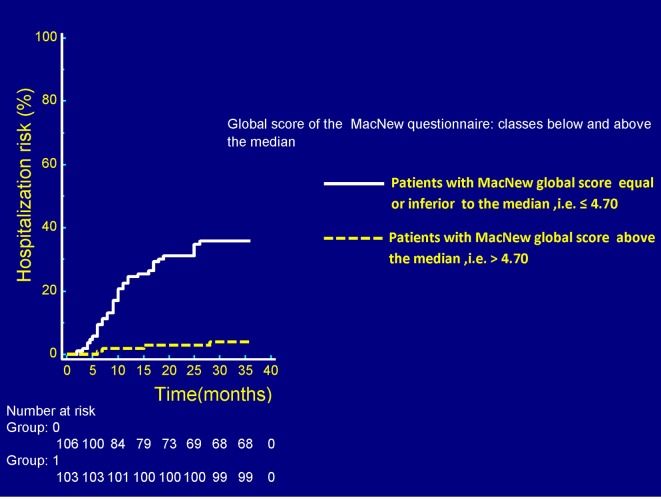
In the graph there is the representation of the respective probabilities of experiencing one or more unscheduled hospitalizations in the patients with MacNew global score equal or inferior to the median (white continuous line) and in those with MacNew global score above the median of the entire population (yellow dashed line). All of the considered patients had undergone a revascularization procedure. Median time delay between revascularization and administration of the questionnaire was 55 days (IQR: 25 - 79 days). A higher (above the median) MacNew global score was associated with a significantly decreased risk of new unplanned hospitalizations over a follow-up of 36 months. P < 0.0001 (log rank test). IQR: interquartile range.

Kaplan-Meier curves were built (Fig. 1) to compare the respective rehospitalization rates between the two groups created according to the MacNew median value found in the patient population: low/moderate (i.e., below the median, namely below the threshold value of 4.70) and high (i.e., above the median) MacNew global score groups. Similarly, unadjusted and adjusted Cox proportional hazards regression models were built to ascertain whether a high MacNew global score inferred by the patient’s responses to the questionnaire was a significant predictor of decreased risk of rehospitalization in patients who had undergone a revascularization procedure (percutaneous or surgical). In this regard, for univariate analysis (Table 1), a high value (i.e., located above the median of the total calculated values of the MacNew score for the entire patient population) on the global score was shown to be significantly associated with a decreased risk of rehospitalization (HR (hazard ratio): 0.4312; 95% CI: 0.3463 - 0.5370; P < 0.0001). After adjustment for age, gender and myocardial infarction as initiating event (Table 2), by incorporating the three above mentioned covariates in a multivariate Cox proportional hazards regression model, the protection exerted by a high MacNew score against the risk of hospitalizations remained significant (HR: 0.0885; 95% CI: 0.0317 - 0.2472; P < 0.0001).

**Table 1:** Univariate Cox Proportional Hazards Regression Analysis

Survival time			Follow-up (months)			
Endpoint			Hospitalization			
Cases summary						
Number of events			42		20.10%	
Number censored			167		79.90%	
Total number of cases			209		100.00%	
Significance level			P < 0.0001			
Coefficients and standard errors						
Covariate	b	SE	P	Exp(b)		95% CI of Exp(b)
Global score	-0.8411	0.1125	< 0. 0001	0.4312		0.3463 - 0.5370

The analysis denotes that as the MacNew global score becomes higher, the risk of hospitalizations significantly decreases. So, a relatively high MacNew global score is associated with a reduced risk of re-hospitalizations (protective association). Exp(b): hazard ratio; CI: confidence interval.

**Table 2:** Multivariate Cox Proportional Hazards Regression Analysis

Survival time			Follow-up (months)		
Endpoint			Hospitalization		
Cases summary					
Number of events			42	20.10%	
Number censored			167	79.90%	
Total number of cases			209	100.00%	
Significance level			P < 0.0001		
Coefficients and standard errors					
Covariate	b	SE	P	Exp(b)	95% CI of Exp(b)
Global score classes above (1) and below (0) the median	-2.4248	0.5267	< 0.0001	0.0885	0.0317 - 0.2472
Age	-0.0007066	0.02654	0.9788	0.9993	0.9489 - 1.0524
History of AMI	-0.4993	0.3169	0.1151	0.6069	0.3272 - 1.1259
Male sex	-0.0324	0.3107	0.9169	0.9681	0.5282 - 1.7744

## Discussion

The administration of the MacNew questionnaire has been proposed by some scholars as a routine investigative tool for patients who have undergone revascularization procedures (either surgical or percutaneous), with the goal of reducing unplanned hospitalizations [3]. In fact in patients with low scores on the questionnaire, the unplanned hospitalizations should be considered more likely and therefore the clinicians should activate all precautions to avoid them, such as critically reviewing and reconsidering the medication dosages or changing the type of molecules that had been originally prescribed. In these cases, a low MacNew global score would serve as a wake-up call that would be able to direct the doctor’s attention on the possibility of concealed procedure-related complications or of greater disease severity or of non-procedural complications [4]. Already since 2007, Pedersen et al [5] showed that a low MacNew global HRQL score was associated with increased risk of major adverse cardiac events (MACE), defined by the occurrence of death, non-fatal MI, CABG or repeat PCI or by a composite of death/non-fatal myocardial infarction but only within the first 6 months subsequent to the revascularization procedure. Furthermore, patient-reported HRQL in patients with CAD is underused in clinical practice even though the limited evidence so far suggests that HRQL is a strong, independent predictor of various health outcomes including rehospitalization [6, 7]. In fact, in 1998, Deaton and colleagues showed a trend for 3-month rehospitalization after CABG in patients exhibiting low HRQL [4]. Almost simultaneously (1998), Lim et al showed that poor HRQL evaluated with the MacNew 6 months after the index event, reliably predicted the occurrence of rehospitalization and mortality as a composite outcome in the 18 months subsequent to HRQL evaluation [6]. In 2010, Schenkeveld et al demonstrated that, measured 1 year after PCI and independent of demographic and clinical characteristics, poor SF-36 health status domain scores, except for the role emotional domain, all were reliable predictors of increased risk of death at 6 years in patients undergoing PCI with drug-eluting stents [8]. However, rehospitalization, a major cause of health care costs [9], was not taken into account as an outcome in these studies. So, in the present report, we have directed our observations on HRQL as a marker of unplanned rehospitalization in patients who had already undergone a revascularization procedure, either PCI or CABG. Thus, considering the results of this study and the other reports in the literature, the choice of including HRQL assessment in routine clinical practice seems very opportune, for better understanding outcomes in patients with cardiovascular disease including prediction of recurrent events after revascularization. The use of disease-specific HRQL questionnaires such as the MacNew can potentially serve as a predictor variable, as a stratifying variable, and as an efficacy outcome in patients with CAD who have experienced a coronary revascularization.

### Study limitations

The outcome data were adjusted only for age, gender and myocardial infarction as the initiating event without taking other potentially meaningful clinical co-variables into account, such as angina, diabetes mellitus, low exercise capacity or psychological disorders. While only baseline HRQL assessment was made in this study, measurement of HRQL changes over time may be a better predictor for adverse outcomes after a revascularization procedure [10].

### Conclusions

A relatively elevated MacNew global score appears to be associated with a significantly decreased risk of new unscheduled hospitalizations after coronary revascularization (either percutaneous or surgical) over a 3-year follow-up. Therefore, the HRQL assessed by using the 27 items of the MacNew questionnaire may be a useful tool, by aiding the physician’s decision making and propitiating a more thoughtful selection of the treatment options suitable for optimizing the management of every individual patient recovering from a surgical (CABG) or percutaneous coronary intervention.

However, the routine use of the MacNew questionnaire for evaluating HRQL needs to be better implemented in clinical practice. This appears to be a desirable goal also considering its potential favorable repercussions on cost-effectiveness. Indeed, our analyses have denoted the capability of predicting the phases of clinical destabilization using the MacNew questionnaire, thereby allowing timely selection of more appropriate drug regimens for patients with CAD who have undergone coronary interventions, in order to prevent unscheduled re-hospitalizations that cause adjunctive costs for the healthcare system.
